# A Non-Autonomous Amphoteric Metal Hydroxide Oscillations and Pattern Formation in Hydrogels

**DOI:** 10.3390/molecules30061323

**Published:** 2025-03-15

**Authors:** Norbert Német, Hugh Shearer Lawson, Masaki Itatani, Federico Rossi, Nobuhiko J. Suematsu, Hiroyuki Kitahata, István Lagzi

**Affiliations:** 1 Department of Physics, Institute of Physics, Budapest University of Technology and Economics, Műegyetem rkp. 3, H-1111 Budapest, Hungary; lawfia5@gmail.com (H.S.L.); masakiitatani.chem@gmail.com (M.I.); 2 Department of Organic Chemistry and Technology, Budapest University of Technology and Economics, Műegyetem rkp. 3, H-1111 Budapest, Hungary; 3 Department of Physical Sciences, Earth and Environment, University of Siena, Piazzetta Enzo Tiezzi 1, 53100 Siena, Italy; federico.rossi2@unisi.it; 4 Meiji Institute of Advanced Study of Mathematical Sciences (MIMS), Meiji University, 4-21-1 Nakano, Tokyo 164-8525, Japan; suematsu@meiji.ac.jp; 5 Graduate School of Advanced Mathematical Sciences, Meiji University, 4-21-1 Nakano, Tokyo 164-8525, Japan; 6 Graduate School of Science, Chiba University, Yayoi-cho 1-33, Inage-ku, Chiba 263-8522, Japan; kitahata@chiba-u.jp; 7 HUN-REN–BME Condensed Matter Physics Research Group, Budapest University of Technology and Economics, H-1111 Budapest, Hungary

**Keywords:** pH oscillation, metal hydroxide, reaction–diffusion, chemical waves

## Abstract

Oscillations in animate and inanimate systems are ubiquitous phenomena driven by sophisticated chemical reaction networks. Non-autonomous chemical oscillators have been designed to mimic oscillatory behavior using programmable syringe pumps. Here, we investigated the non-autonomous oscillations, pattern formation, and front propagation of amphoteric hydroxide (aluminum (III), zinc (II), tin (II), and lead (II)) precipitates under controlled pH conditions. A continuous stirred-tank reactor with modulated inflows of acidic and alkaline solutions generated pH oscillations, leading to periodic precipitation and dissolution of metal hydroxides in time. The generated turbidity oscillations exhibited ion-specific patterns, enabling their characterization through quantitative parameters such as peak width (*W*) and asymmetry (*As*). The study of mixed metal cationic systems showed that turbidity patterns contained signatures of both hydroxides due to the formation of mixed hydroxides and oxyhydroxides. The reaction–diffusion setup in solid hydrogel columns produced spatial precipitation patterns depending on metal cations and their concentrations. Additionally, in the case of tin (II), a propagating precipitation front was observed in a thin precipitation layer. These findings provide new insights into precipitation pattern formation and open avenues for metal ion identification and further exploration of complex reaction–diffusion systems.

## 1. Introduction

Chemical oscillators are chemical kinetic systems in which the concentration of the chemical species shows oscillatory behavior in time. Oscillations in these systems can be maintained either in batches (e.g., the Briggs–Rauscher [1,2,3] and the Belousov–Zhabotinsky [4,5,6] (BZ) reactions) or open systems (e.g., formaldehyde-sulfite-gluconolactone [7] and hydrogen peroxide-sulfite [8]). Experiments in open chemical systems have been realized in continuous stirred tank reactors (CSTRs), which contribute to maintaining the chemical systems far from their thermodynamic equilibria. In autonomous chemical oscillators, emergent phenomena occur due to the interplay of chemical reaction subnetworks. Various phenomena can be designed and observed in the CSTRs, such as oscillations, bistability, and chemical chaos [6,9,10,11,12,13,14]. Several attempts have been made to control chaos in open chemical systems, showing the power of chemical system engineering [6,14]. Recently, the focus of investigations of oscillatory systems moved from the individual to the system chemistry level, studying the behavior of a set of connected CSTRs [15].

These chemical reactions can be coupled with the diffusion and advection of the chemical species to generate complex patterns. The existence of mass transport enables the generation of dynamic and stationary patterns [16] and induces chemical oscillations in otherwise non-oscillating chemical systems [17]. These experiments can be performed in a thin liquid layer or gel matrix. Emblematic examples of the dynamic patterns in reaction–diffusion systems are autocatalytic front propagation [18,19] and the formation of spiral waves and target patterns in the BZ reaction [18]. Turing patterns and precipitation structures (including periodic precipitation) fall into stationary patterns [19,20,21,22,23,24].

In contrast, non-autonomous oscillatory systems can be designed using periodic changes in experimental conditions. For instance, non-autonomous pH oscillations in a CSTR were successfully engineered using a sinusoidally modulated flow rate of both the acid solutions and base in antiphase using two programmable syringe pumps. In the past four years, this strategy has been used to couple self-assembly (including oleic acid vesicles, gold nanoparticles [25], metal–organic framework building blocks [26]) with non-autonomous pH oscillation systems.

Most metal hydroxides form insoluble precipitation in water, such as Al(OH)_3_, Zn(OH)_2_, Cu(OH)_2_, and Ni(OH)_2_ [27,28,29,30]:

$${Me}^{n+}+n\;{OH}^{-}⇌{Me(OH)}_{n}\;↓\;n=1,2,3,4$$

However, a few transition metals can form amphoteric hydroxides, including aluminum (III), zinc (II), tin (II), and lead (II) ions, which can dissolve into a solution under strong acid and base conditions due to the reversible precipitation and complex formation [31,32,33,34]. Metal hydroxides are typically strong bases, which means the reaction with a strong acid would follow an acid–base neutralization reaction and form water and salt. While amphoteric metal hydroxides in the presence of a high excess strong base such as sodium or potassium hydroxides can form water-soluble hydroxo complexes:

$${Me(OH)}_{n}+m\;{OH}^{-}⇌{Me(OH)}_{n+m}^{m-}\;n=2,3\;and\;m=1,2$$

The solubility of metal hydroxide depends on the pH. Metal hydroxide formation is also affected by the metal salt used (e.g., during SnCl_2_ hydrolysis, Sn(OH)Cl white precipitation also appears; this is why a minimum amount of acid should be added to the solution to obtain a transparent solution).

Recent trends in the study of oscillatory reactions have been limited to designing oscillatory chemical networks and investigating how they can be used in controlling oscillatory-dependent (e.g., pH- and redox-dependent) processes; for instance, using them in soft robotics [35,36,37]. BZ reaction was also applied in the material design, generating spherulitic crystals [38], controlling the radical polymerization of acrylonitrile [39,40], and synthesis of giant polymeric vesicles [41]. Practical applications of oscillatory reactions in analytical chemistry are limited [42]. In this paper, we introduced experiments investigating the precipitation and dissolution (due to complex formation) processes of different amphoteric metal hydroxides in a non-autonomous pH oscillatory system. The pH change was driven with antiphase inflows of acid and base solutions in a CSTR using two programmable syringe pumps (Figure S1). Periodic pH changes generated oscillation in the turbidity of the solution due to the precipitation and complex formation of the formed hydroxides. We characterize the produced periodic turbidity patterns in time by calculating the width and asymmetry of the peaks. Each amphoteric metal salt generated different time patterns, which can be used to identify the cations. This idea was extended in a reaction–diffusion setup, where the metal ions diluted with strong acid were homogeneously distributed in a solid hydrogel, and the strong base was diffused from outside, generating precipitation patterns in the gel matrix. Finally, we observed a front propagation in a thin layer of the tin (II) hydroxide precipitation layer.

## 2. Results

### 2.1. Non-Autonomous Oscillations of Amphoteric Hydroxides (Al^3+^, Zn^2+^, Sn^2+^, and Pb^2+^)

**Figure 1:**
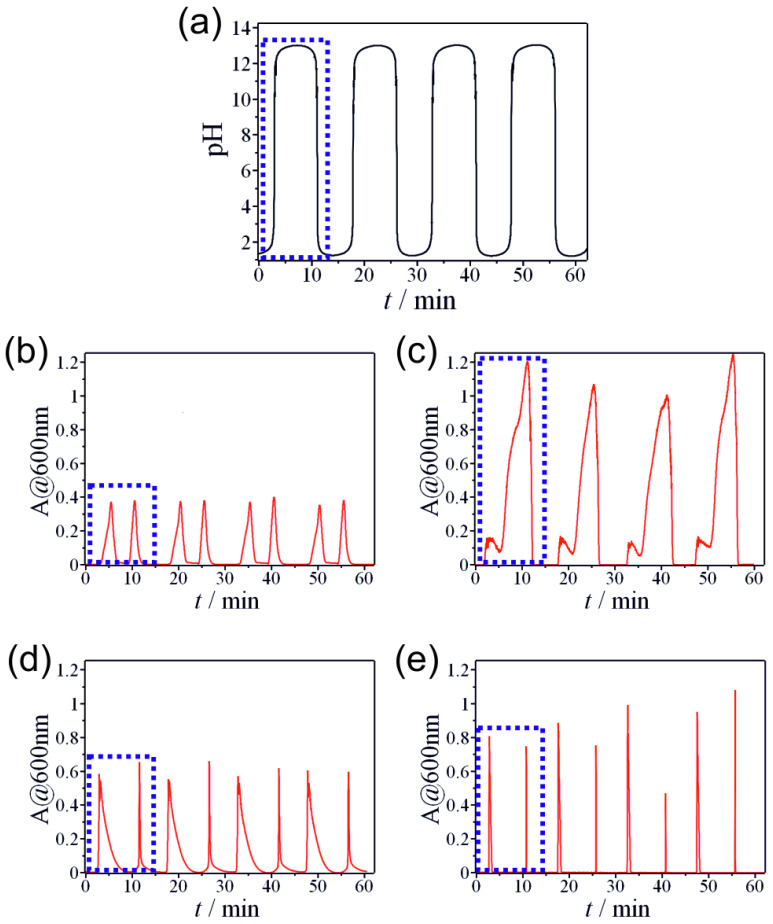
(**a**) Generated non-autonomous pH oscillations in the CSTR due to the sinusoidally modulated antiphase inflows of acid and base ([HCl]_0_ = 300.0 mM and [NaOH]_0_ = 300.0 mM). Turbidity oscillations of individual metal hydroxides in the CSTR using a non-autonomous pH oscillator: (**b**) aluminum (III) ([Al^3+^]_0_ = 20.0 mM); (**c**) zinc (II) ([Zn^2+^]_0_ = 6.0 mM); (**d**) tin (II) ([Sn^2+^]_0_ = 4.0 mM); (**e**) lead (II) ([Pb^2+^]_0_ = 0.5 mM). The dotted line boxes show one-cycle pH oscillation (**a**) and corresponding double-peak turbidity oscillations (**b**–**e**).

In a typical experiment of non-autonomous pH oscillations, we made two solutions: 300.0 mM hydrochloric acid and 300.0 mM sodium hydroxide. Both aqueous solutions contained metal salt individually or as different mixtures. In the acidic solutions, metals were present as free cations, while in the alkaline solutions, as hydroxo complexes, both stock solutions were transparent. The metal contents were the same in both stock solutions. Thus, no dilution occurred during the oscillation process in the CSTR. The solutions were simultaneously introduced into the reactor, which was initially filled with distilled water, with a modulated flow rate in the antiphase in time using two programmable syringe pumps. The CSTR was a quartz cuvette placed in a UV-vis spectrophotometer. The metal hydroxide precipitation was monitored by recording the turbidity of the solution (Figure S1). It should be noted that turbidity was affected by the amount and the quality (average size and dispersity) of the metal hydroxide and oxyhydroxide colloid particles. We applied a sinusoidal waveform function of the flow rate of the solutions of the reagents. Since one of the pumps contained the acidic solution, while another the basic solution, the antiphase flow rates in time generated a series of switching between acidic and alkaline conditions (Figure 1a). This setup was already successfully used in several of our previous research studies [25,26]. The switching process occurred following the periodical change of the pH environment and the consequent appearance of a double-peaked signal in the turbidity of the solution due to the precipitation of hydroxides. Figure 1b–e shows the turbidity oscillations of different individual metal precipitations in the CSTR. The pH oscillation range was between 1 and 13 in all cases, while the turbidity curves exhibited different characteristic behaviors. However, the turbidity temporal patterns shared the same characteristics in all cases. Namely, one pH cycle generated two cycles of turbidity change. When the pH increased, it caused the formation of white precipitate in the CSTR (inducing an increase in turbidity, with the transparent solution becoming turbid) followed by its dissolution due to the hydroxo complex formation (inducing a decrease in turbidity, with the solution becoming transparent again). When the pH reached its maximum and decreased, the precipitate appeared again in the reactor and later dissolved due to the reversible nature of the precipitation and hydroxo complex formation.

Each metal cation generated different temporal turbidity patterns, which can be used as a fingerprint of the metal cations. In the case of aluminum (III) and lead (II) (Figure 1a,d), the peaks corresponding to the increasing (pH ↑) and decreasing (pH ↓) branches of the pH were almost identical. Only a slight difference can be noticed; namely, the turbidity signal corresponding to the branch of pH ↑ was slightly wider for both metal cations. However, in the case of zinc (II) and tin (II) (Figure 1b,c), the two peaks corresponding to pH ↑ and pH ↓ branches showed a significant difference. Two arguments can explain this phenomenon. First, the turbidity depends on the amount, average particle size, and dispersity of the precipitate particles. Secondly, various metal cations can produce different polymorphs of hydroxides and oxyhydroxides [43,44,45].

**Figure 2:**
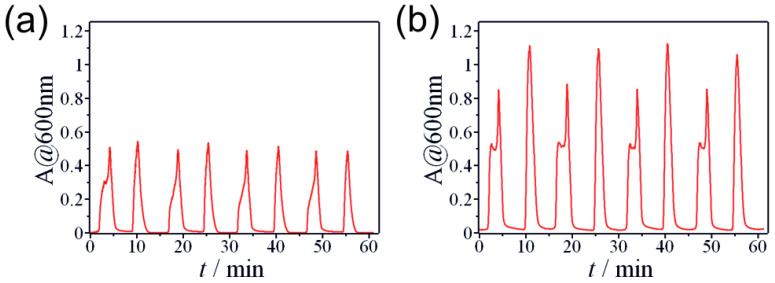
Turbidity oscillations of aluminum (III) and tin (II) metal hydroxide mixtures generated in the CSTR by a non-autonomous pH oscillator ([HCl]_0_ = 300.0 mM and [NaOH]_0_ = 300.0 mM): (**a**) [Al^3+^]_0_ = 20.0 mM and [Sn^2+^]_0_ = 2.0 mM; (**b**) [Al^3+^]_0_ = 20.0 mM and [Sn^2+^]_0_ = 4.0 mM.

After investigating individual metal cations, we also studied the controlled precipitation of mixed metal cations. Figure 2 presents an oscillation pattern obtained in the case of tin (II) and aluminum (III) ions while applying different concentration ratios. Changing the ratio of the two metal cations resulted in different intensities of the turbidity peaks. According to our results, we can recognize both the tin (II) and aluminum (III) characteristics in the absorbance curves, especially at the highest concentration (Figure 2b). The peak corresponding to the pH ↑ branch has a double-peak structure originating from the broader pH ↑ peak of tin (II) (Figure 1c). It should be noted that the precipitate contained not only individual tin (II) and aluminum (III) hydroxides but also mixed hydroxides (Al_x_Sn_y_(OH)_(3x+2y)_). The same argument can be applied in the case of the mixtures of other two or three metal cations (Figure S2).

We introduced the following physical quantities to characterize quantitatively the produced turbidity peaks of the various hydroxides:

(1) $$W=\sqrt{\langle t^{2}\rangle }={\left(\frac{\int_{t_{s}}^{t_{e}}t^{2}u\left(t\right)dt}{\int_{t_{s}}^{t_{e}}u\left(t\right)dt}\right)}^{1/2},$$

where W is the characteristic width of the peak, and $u\left(t\right)$, $t_{s}$, and $t_{e}$ are the turbidity signal, the start, and the end of the peak in time, respectively. The time is defined so that *t* = 0 is at turbidity maximum for each peak. The second physical quantity is the asymmetry of the peak (As), which is:

(2) $$As=\frac{\langle t\rangle }{W}=\frac{1}{W}\int_{t_{s}}^{t_{e}}tu\left(t\right)dt.$$

**Figure 3:**
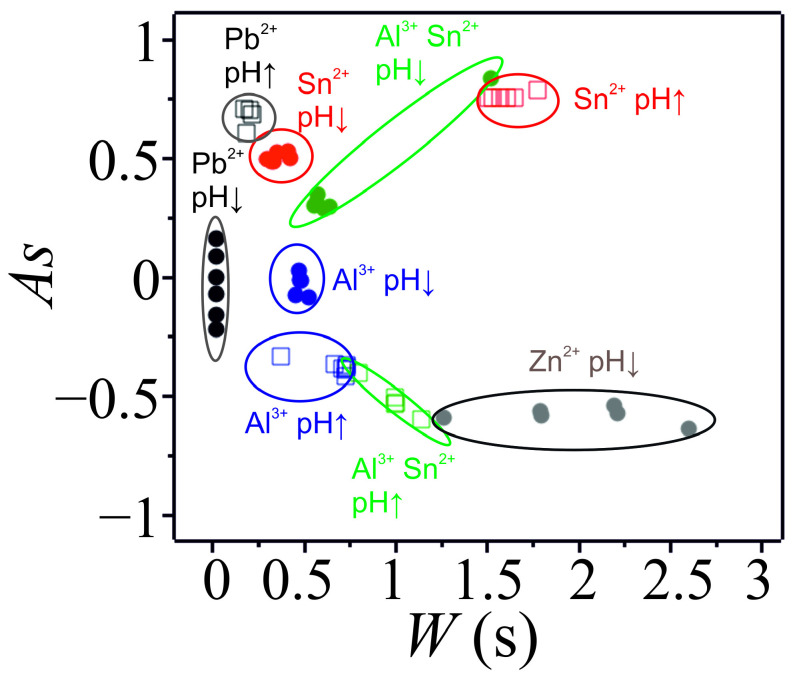
Scatter plot of the asymmetry (*As*) and width (*W*) of the turbidity peaks in aluminum (III) ([Al^3+^]_0_ = 20.0 mM), zinc (II) ([Zn^2+^]_0_ = 5.0 mM), tin (II) ([Sn^2+^]_0_ = 4.0 mM), and lead (II) ([Pb^2+^]_0_ = 0.5 mM), as well as mixed aluminum (III) and tin (II) ([Al^3+^]_0_ = 20.0 mM and [Sn^2+^]_0_ = 4.0 mM) hydroxide systems. Symbols pH↑ and pH↓ indicate data corresponding to the increasing and decreasing branches of the pH, respectively.

Figure S3 shows the determination of the physical quantities characterizing the turbidity peaks. Figure S4 presents the calculated W and A for the individual metal cations and the mixture of aluminum (III) and tin (II). Each metal cation has distinguishable characteristic values of W and As corresponding to pH ↑ and pH ↓ branches. To highlight this feature, we prepared the scatter plot of W and As (Figure 3). It can be seen that individual cations and the mixture created well-separated clusters. In this way, the pH oscillation-driven hydroxide formation can be used as a quantitative analytical tool to determine the cation in a solution. In addition, this method can be applied to mixtures of different metal cations.

**Figure 4:**
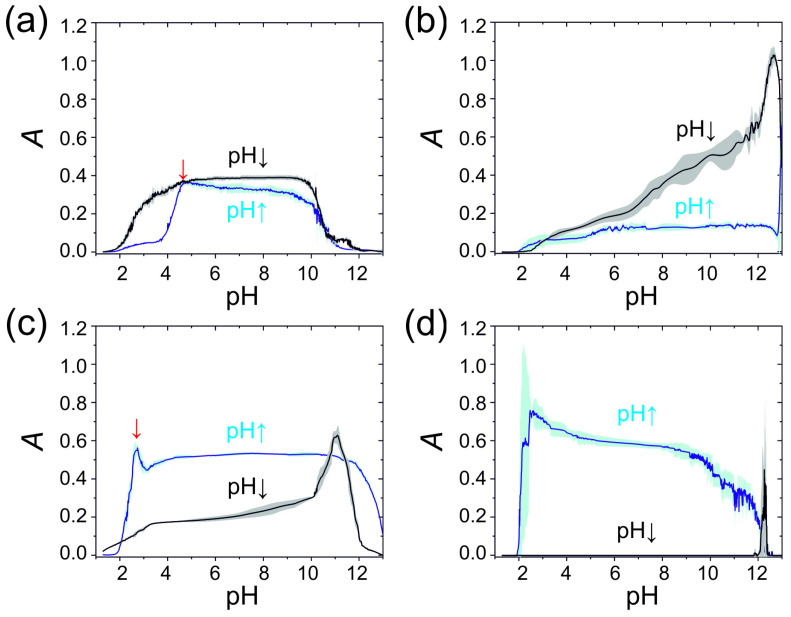
Dependence of the turbidity on pH corresponding to pH ↑ and pH ↓ branches with standard deviations generated by non-autonomous pH oscillations in the CSTR due to the sinusoidally modulated antiphase inflows of acid and base ([HCl]_0_ = 300.0 mM and [NaOH]_0_ = 300.0 mM): (**a**) aluminum (III) (the turbidity data are from Figure 1b, [Al^3+^]_0_ = 20.0 mM); (**b**) zinc (II) (the turbidity data are from Figure 1c, [Zn^2+^]_0_ = 6.0 mM); (**c**) tin (II) (the turbidity data are from Figure 1d, [Sn^2+^]_0_ = 4.0 mM); (**d**) lead (II) (the turbidity data are from Figure 1e, [Pb^2+^]_0_ = 0.5 mM). Red arrows indicate in (**a**,**c**) the maximum turbidity in the pH ↑ branch.

We introduced an additional method to identify cations based on the turbidity versus pH curves generated from the pH in both time and turbidity in time measurement curves. Each cation investigated has characteristic curves corresponding to pH ↑ and pH ↓ branches (Figure 4). Figure 4 also shows the error band (standard deviation) propagation in individual cationic systems for four cycles. One can see that the standard deviation of the data is slight and there is no hysteresis effect, indicating the precipitation and complex formation were thermodynamically controlled by pH.

**Figure 5:**
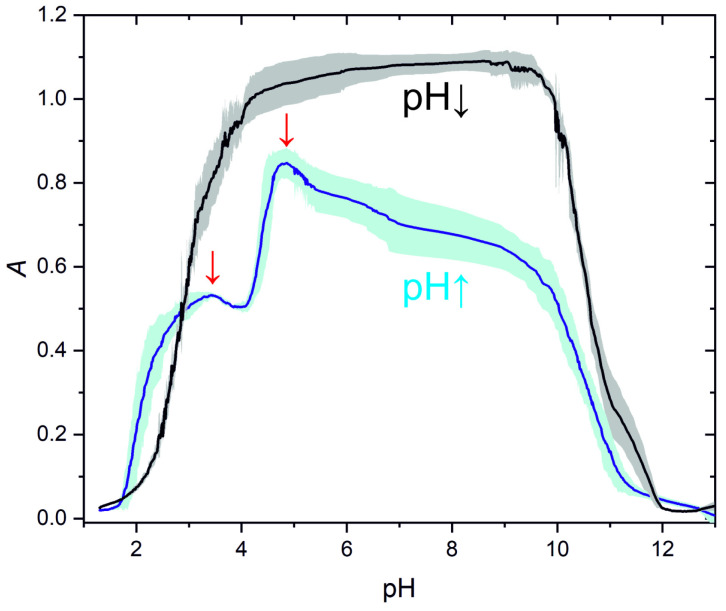
Dependence of the turbidity on pH corresponding to pH ↑ and pH ↓ branches with standard deviations generated in a mixed cationic system by non-autonomous pH oscillations in the CSTR due to the sinusoidally modulated antiphase inflows of acid and base ([HCl]_0_ = 300.0 mM and [NaOH]_0_ = 300.0 mM): [Al^3+^]_0_ = 20.0 mM and [Sn^2+^]_0_ = 4.0 mM (the turbidity data are from Figure 2b. Red arrows indicate the maxima of the turbidity in the pH ↑ branch.

We also plotted the turbidity in time curves for two branches of the aluminum (III): tin (II) mixed system (Figure 5). The plot shows that the pH ↓ branch was reminiscent of the aluminum (III) pH ↓ branch (Figure 4a). However, in the pH ↑ branch, we can recognize two maxima (at pH ~3.5 and ~5) corresponding to the global maxima of the individual aluminum (III) and tin (II) pH ↓ branches (Figure 4a,c). It should be noted that there is a half-pH unit shift in the position of the maxima in the mixed system compared to the individual cationic systems. In this way, we presented two approaches that can be used to identify mixed cationic systems.

To demonstrate the potential power of non-autonomous pH oscillation, we were also able to create non-autonomous turbidity oscillations in copper (II) and zinc (II) systems using an ammonia solution as the base (Figure S5). In this case, the complex formation consisted of two main routes: the formation of hydroxo and ammine complexes.

### 2.2. Pattern Formation in a Hydrogel Column

**Figure 6:**
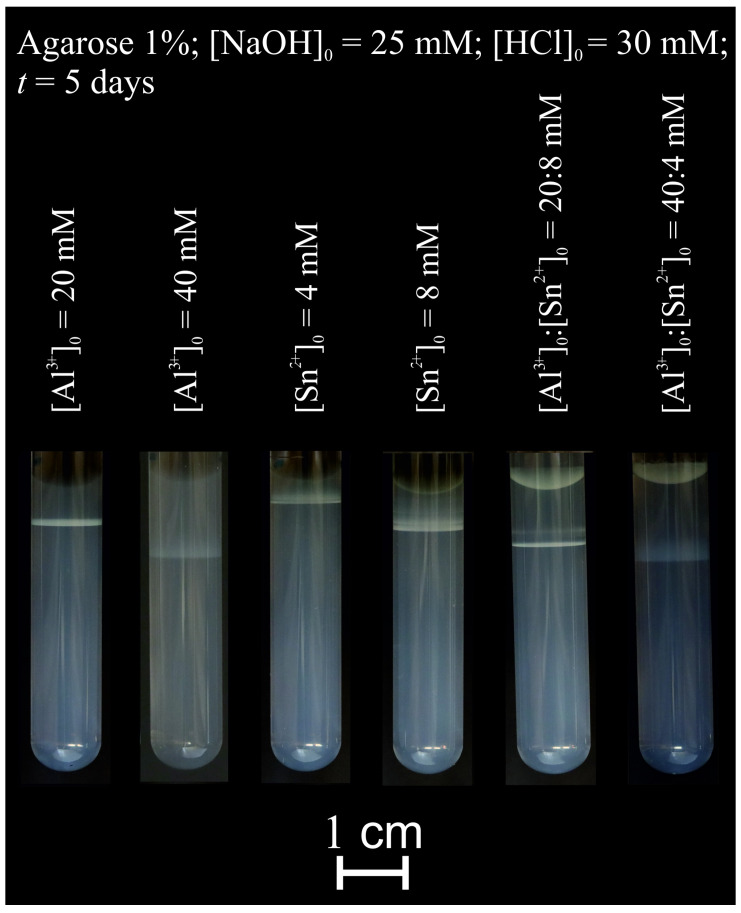
Photographs of aluminum (III) and tin (II) metal hydroxide patterns in agarose gel (1% *w*/*w*) with different metal concentrations and ratios after 5 days. Initially, the gel contained 50.0 mM sodium hydroxide. After gelation, a solution containing 30.0 mM hydrochloric acid and transition metal cations (or their mixtures) was layered on top of the gel columns.

We also investigated the precipitation of amphoteric hydroxides in a solid-phase gel medium. In a typical experiment, we solidified agarose gel in the presence of sodium hydroxide in test tubes and layered hydrochloric acid containing metal salt solution on the top of the gel. We waited until white precipitation bands were formed in the gel matrix. As mentioned earlier, in the case of tin (II) (which could also form white precipitate under neutral pH conditions in water), the acidic environment was necessary to set metal salts as free cations in the outer electrolyte. In this reaction–diffusion setup, the white precipitation bands appeared in different gel parts according to the metal quality and concentration. At the beginning of the experiments, a white precipitation zone appeared at the liquid–gel interface. Later, this moved farther from the interface due to the precipitation and dissolution of the precipitate (at acidic pH). Figure 6 shows the generated hydroxide patterns in the agarose gel. The position of the zones at the given time depended on the quality of the metal cations and their concentrations. Higher concentrations of the metal cations in the outer electrolytes further formed the precipitation zones measured from the liquid–gel interface. This can be attributed to higher concentrations generating enhanced diffusion flux of the metal ions in the gel matrix. In the case of a mixture of the cations, we could observe complex structures consisting of two zones (Figure 6). A similar setup was used in recent works, in which the separation of several metal ions based on the coupling of ion diffusion and precipitation kinetics was presented. In these studies, a feedstock solution of dissolved battery electrodes was placed on top of a solid hydrogel column loaded with a precipitating agent (sodium hydroxide). As the cations diffused into the gel, after diffusion and reaction, a gradient of precipitates formed along the length of the gel reactor. [46,47] Further test tube experiments with other metal ion mixtures can be found in Figure S6. Each type of metal cation usually generated a single precipitation band in the gel column. However, their mixtures (Al^3+^, Zn^2+^, Sn^2+^, and Pb^2+^) produced complex bended structures. This pattern formation might be attributed to the formation of mixed hydroxides and oxyhydroxides.

### 2.3. Propagation of a Precipitation Front in a Thin Layer of Tin (II) Hydroxide Precipitate

Pattern formation in reaction–diffusion systems provides intriguing examples of the emergence of macroscopic order from molecular reaction events and Brownian motion. As discussed in the Introduction, these patterns can be categorized as being spatially steady or unsteady and temporally static or dynamic. An extensively studied area of non-linear chemistry is wave propagation in an excitable media. The BZ reaction is a typical example of investigating wave propagation and other related phenomena (excitability, bistability, and chaos). The periodic precipitation (Liesegang phenomenon) occurs when a precipitation reaction is coupled to the mass transport (usually diffusion) of reagents in solid hydrogels, generating a series of precipitation zones [48]. In the past, it has been accepted that there is no dynamic pattern formation in precipitation systems, and there have been no similarities between patterns in excitable media and patterns emerging in precipitation reactions. This is because precipitation systems are relatively simple, static, and usually contain only two inorganic salts, and the formation of the precipitate is mainly dominated by local nucleation and growth processes. It is hard to imagine that dynamic waves can exist in precipitation (heterogeneous) systems similar to those in excitable (homogeneous) systems. Recently, it was demonstrated that similar self-organized chemical waves can exist in several precipitation reactions. The spontaneous appearance of traveling waves (spirals and target patterns) inside a thin and moving precipitation layer in a hydrogel medium was reported in aluminum hydroxide, zinc hydroxide, and mercuric iodide precipitation systems [49,50,51,52,53]. In this setup, the wave propagation occurred perpendicular to the diffusion flux of the outer electrolyte (hydroxide solution), and the coexistence of a moving precipitation layer with traveling waves inside this precipitation layer was observed. Until now, only three precipitation systems with precipitation and complex formation exhibited this behavior.

**Figure 7:**
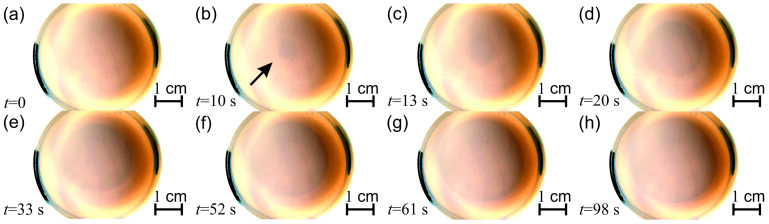
Front propagation of tin (II) hydroxide precipitation system. The solid agarose gel (0.5% *w*/*w*) contained 30.0 mM of tin (II), and a sodium hydroxide solution of 2.500 M was layered on the top of the gel in the Petri dish. After ~10 min, the emergence of a precipitation front was observed, which moved downward in the gel disk. *t* = 0 corresponds to the time when the front propagation started. The black arrow (panel (**b**)) shows the appearance of the precipitation front, which evolves from (**a**–**h**).

**Figure 8:**
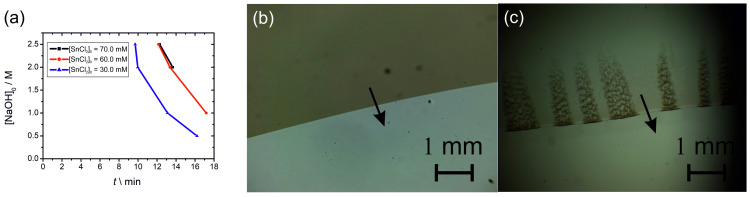
The appearance time (induction time) of the precipitation front at various concentrations of sodium hydroxide (outer electrolyte) and tin (II) chloride (inner electrolyte) (**a**). Propagation of the precipitation zone in (**b**) agarose hydrogel (1% *w*/*w*) with a thickness of 70 μm and (**c**) confined liquid layer with the same thickness, respectively. The black arrow shows the propagation of the precipitation zone. The solid agarose gel and liquid layer in the HS cell contained 30.0 mM of tin (II), and a sodium hydroxide solution of 2.500 M was used as an outer electrolyte.

In typical experiments, the tin (II) cations were homogeneously distributed in an agarose gel. A solution of sodium hydroxide (0.5, 1.0, 2.0, and 2.5 M) was used as the outer electrolyte placed on top of the gel in the Petri dish. The hydroxide ions diffused into the gel matrix, and precipitation reaction with tin (II) ions produced a white, insoluble precipitation layer at the gel interface (Sn^2+^ (aq) + 2 OH^−^ (aq) → Sn(OH)_2_ (s)). Subsequently, this layer moved due to the complex formation (dissolution) of tin hydroxide in excess hydroxide ions (outer electrolyte), producing a soluble hydroxo complex (Sn(OH)_2_ (s) + 2 OH^−^ (aq) → SnOH_4_^2−^ (aq)). Due to these phenomena, a thin precipitation layer advanced in the gel driven by the diffusion of the chemical species. It should be noted that in forming the precipitate, not only the tin (II) hydroxide in its pure form could be considered but other non-water-soluble forms of tin (II) hydroxides and oxyhydroxides might form in the process. The thickness of the precipitation layer ranged between 10 and 100 μm depending on time and the concentration of tin (II). After ~10 min, the precipitation front with an average speed of ~0.2 mm/s was observed perpendicular to the planar chemical front of the outer electrolyte moving downward in the gel disk (in the Petri dish, Figure 7). The front propagated from the center part towards the edge of the Petri dish. In contrast to the previously discovered systems, the front propagation started later than in aluminum (III) hydroxide, zinc (II) hydroxide, and mercury (II) iodide systems [49,50,51,52,53]. In these chemical systems, the pattern formation occurred a few minutes after the initialization of the reaction. Another difference is that in the tin (II) hydroxide, we observed only the front instead of the waves (target pattern, spirals, and double spirals). The third difference is that the generated waves propagated with a superdiffusive nature in the aluminum (III) hydroxide and mercury (II) iodide systems [52]. Interestingly, the analysis did not show a superdiffusive nature in the tin (II) hydroxide system (Figure S7). The slope of the log–log plot of the position of the front versus time for longer times was 0.30, indicating subdiffusive nature. However, this investigation requires further effort in the future. The induction period of the front propagation (the time elapsed from the start of the experiment till the appearance of the front) depended on the concentrations of the inner (tin (II)) and outer (sodium hydroxide) electrolytes. A decrease in the tin (II) and an increase in the sodium hydroxide concentrations generated a shorter induction time (Figure 8a). Interestingly, we did not find either front of wave propagation in the lead (II) hydroxide system. 

The evolution of the precipitation zone in the tin (II) system was investigated in a thin gel and pure liquid film. The usual argument for applying the gelled medium is that it prevents the sedimentation of the formed particles, thus inhibiting hydrodynamic effects. Some studies highlighted the effect of the gel to generate periodic precipitation providing heterogeneous nucleation centers [54]. In all studies conducted since the discovery of this type of pattern formation [55,56,57], solid hydrogels (mostly gelatin, agarose, agar-agar, polyvinyl alcohol, and polyacrylamide) have been used [58,59,60,61,62,63,64,65,66,67]. Recently, it has been presented that periodic precipitation can be formed in a confined thin liquid film (with a thickness of ~70 µm) using a Hele–Shaw (HS) cell [68]. In this setup, a thin metal wire separated the two solutions containing precipitating ions. When the wire was removed, the precipitation started due to the diffusion and precipitation of the chemical species. This result highlighted the universality of the Liesegang phenomenon. We found a difference in the structures of the precipitation zones formed in gelled and liquid media. In the gel matrix, the precipitation zone was homogeneous and intact (Figure 8b). However, in a pure liquid phase, we observed the formation and evolution of precipitation segments (Figure 8c and Video S1). The nature and mechanism of this new type of pattern formation are unknown and beyond the scope of this contribution. However, a detailed investigation should be conducted to reveal the possible mechanism.

## 3. Methods

### 3.1. Reagents

We used the following reagent-grade chemicals: hydrochloric acid solution (32% HCl, *ρ* = 1.16 g/mL, Sigma-Aldrich, Burlington, MA, USA), sodium hydroxide (NaOH, reagent grade ≥ 98%, Sigma-Aldrich, Burlington, MA, USA), anhydrous aluminum (III) chloride (AlCl_3_, reagent grade ≥ 98%, Sigma-Aldrich, Burlington, MA, USA), zinc (II) chloride (ZnCl_2_, reagent grade ≥ 96%, Sigma-Aldrich, Burlington, MA, USA), tin (II) chloride (SnCl_2_, reagent grade ≥ 98%, Sigma-Aldrich, Burlington, MA, USA), and lead (II) nitrate (Pb(NO_3_)_2_, reagent grade ≥ 99%, Reanal, Budapest, Hungary). We used Agarose Type I-A low EEO (Sigma-Aldrich, Burlington, MA, USA) for the reaction–diffusion experiments. All chemicals were used without further purification.

### 3.2. Non-Autonomous Metal Hydroxide Oscillators

In this experiment series, the acid–base neutralization reactions containing different transition metals were carried out in CSTR with a volume of 8.5 mL and stirring rate of 800 rpm at room temperature (24 ± 0.5) °C. All the metal salts and their different mixtures were dissolved both in the acid and base solutions ([HCl]_0_ = [NaOH]_0_ = 300.0 mM) with the same concentrations. The acid and alkaline solutions were allowed to flow simultaneously with the sinusoidally modulated flow rate into the CSTR using two programmable syringe pumps. We used syringe pumps instead of peristaltic pumps to minimize fluctuations in the flow rate and provide a continuous and stable reagent flow in the CSTR. We used the sinusoidal inflow rate function:

$$r_{acid}\left(t\right)=r_{0}+r_{A}\sin\left(\frac{2\pi }{T}t\right),$$

$$r_{base}\left(t\right)=r_{0}+r_{A}\sin\left(\frac{2\pi }{T}t+\varphi \right),$$

where $r_{acid}$ and $r_{base}$ are the modulated inflow rates of the acidic and alkaline solutions, respectively. *T* = 15 min is the time period of the waveform, and *r*_0_ was 15 µL/s. In experiments, we used antiphase inflow rates for acidic and alkaline solutions (i.e., the phase difference of *φ* = π was applied). There was also a third (peristaltic) pump for outflow. The pH in CSTR was monitored by a pH microelectrode (Mettler Toledo Lab pH Electrode LE422, Mettler Toledo, Columbus, OH, USA) connected to an amplifier and a multimeter. Before the experiments, we performed a 3-point calibration using buffers. Turbidity change was monitored in kinetic mode by a VWR UV-1600 PC spectrophotometer (VWR International, Radnor, PA, USA) at *λ* = 600 nm wavelength (in the case of zinc (II), aluminum (III), tin (II), and lead (II) ions and their mixtures).

### 3.3. Formation of Layered Metal Hydroxide in a Hydrogel

First, we prepared a solution of 50.0 mM sodium hydroxide. After 30 min, 2% *w*/*w* agarose gel was prepared by mixing agarose powder in distilled water, and the mixture was heated to 80 °C until the agarose was completely dissolved. The hot agarose solution was mixed with the sodium hydroxide solution in a 1:1 ratio and stirred to obtain a homogeneous solution ([NaOH]_0_ = 25.0 mM with 1% *w*/*w* agarose concentration). Then, the mixture was poured into test tubes (with a length and inner diameter of 7.5 cm and 1 cm, respectively), filling two-thirds of the total volume. After the gelation process (24 h), a solution containing 30.0 mM hydrochloric acid and transition metal cations (or their mixtures) was layered on top of the gel columns. The pattern formation in the gels was monitored for five days. All experiments were performed at room temperature (24 ± 0.5) °C (Table 1 and Table 2).

### 3.4. Front Propagation in Tin (II) Chloride System

In the study of precipitation front propagation, 0.5% *w*/*w* of agarose gel was produced as described in the previous section, with the addition of different concentrations of the tin (II) chloride in a hydrogen chloride solution (acid was used to prevent the precipitation of tin (II) ions in solution). A 10 mL hot agarose gel solution containing tin (II) chloride was poured into a Petri dish 5 cm in diameter and cooled to facilitate hydrogel formation. After cooling down to room temperature, 5 mL of a sodium hydroxide solution (0.500, 1.000, 2.000, and 2.500 M) was poured onto the hydrogel layer, and the resulting precipitation reactions were observed by Canon 90D (Canon, Tokyo, Japan) with a macro-objective (Canon EF-S 60 mm f/2.8 macro USM lens, Canon, Tokyo, Japan).

Front propagation experiments were also carried out in a thin liquid film (two-dimensional environment) confined to an HS cell. The experiments used a commercially available HS cell with a vertical gap distance of 70 µm. The setup involved placing a basic solution outside the HS cell window in contact with a tin (II) chloride solution confined to the HS cell at the edge of the cell window. The pattern formation was monitored by a Visiscope TL324H microscope with an objective lens of 10× (VWR International, Radnor, PA, USA). All experiments were performed at room temperature (24 ± 0.5) °C.

## 4. Conclusions

In this study, we explored the non-autonomous oscillatory behavior of amphoteric hydroxides, their pattern formation in hydrogel columns, and the propagation of precipitation fronts in tin (II) hydroxide systems. Our findings highlight the distinct turbidity oscillation patterns of different metal hydroxides under periodic pH modulation, demonstrating that each metal cation produces a unique fingerprint. These patterns were further analyzed through the characteristic width (*W*) and asymmetry (*As*) of turbidity peaks, showing well-separated clusters in scatter plots, suggesting the potential for analytical applications in metal ion detection. A similar idea was employed based on temporal pattern analysis to identify organic solvents. The time dependence of the ohmic resistance of wetted mesoporous multiwall carbon nanotube films by organic solvents was investigated. The generated resistance–time profiles (evaporation patterns) were used to analyze the used organic solvents qualitatively [69]. The growing impact and interest in machine learning and artificial intelligence can help analyze temporal patterns and identify cations even in multicomponent systems [70].

The investigation of mixed metal cationic systems revealed that turbidity patterns contained signatures from both metal hydroxides due to the formation of mixed hydroxides and oxyhydroxides. This provides insights into the controlled co-precipitation of metal cations and their potential applications in material synthesis and analytical chemistry.

In a reaction–diffusion setup, we demonstrated the formation of precipitation zones in agarose gels, where the spatial arrangement depended on the type of metal cation and concentration. Higher concentrations resulted in bands appearing farther from the liquid–gel interface due to enhanced diffusion flux. Mixtures of metal cations led to the emergence of complex banded structures, suggesting interactions between the kinetics of the formation of hydroxides in the gel matrix.

Finally, we observed the propagation of precipitation fronts in tin (II) hydroxide systems. The advance of the precipitation zone was driven by hydroxide ion diffusion, precipitation, and subsequent complex formation. Unlike previously studied precipitation reactions, this system exhibited only front propagation rather than wave patterns. The induction period was influenced by the concentrations of inner (tin (II)) and outer (sodium hydroxide) electrolytes, with shorter induction times observed at higher sodium hydroxide concentrations. Additionally, differences between gelled and liquid-phase systems were noted, with the liquid-phase experiment revealing segmental precipitation patterns.

Overall, our findings can contribute to a deeper understanding of oscillatory precipitation phenomena, reaction–diffusion pattern formation, and front propagation in precipitation systems. These results open new avenues for using pH-driven precipitation as an analytical tool and for further studies on dynamic pattern formation in inorganic systems.

## Figures and Tables

**Table 1:** The experimental conditions for the formation of the hydroxide pattern in a hydrogel column presented in Figure 4.

Agarose 1%; [NaOH]_0_ = 25 mM; [HCl]_0_ = 30 mM; *t* = 5 Days			
Vial	[Al^3+^]_0_/mM	[Sn^2+^]_0_/mM	Ratio
1	20.0	-	-
2	40.0	-	-
3	-	4.0	-
4	-	8.0	-
5	20.0	8.0	5:2
6	40.0	4.0	10:1

**Table 2:** The experimental conditions for the formation of the hydroxide pattern in a hydrogel column presented in Figure S6.

Agarose 1%; [NaOH]_0_ = 25 mM; [HCl]_0_ = 30 mM; *t* = 7 Days					
Vial	[Al^3+^]_0_/mM	[Zn^2+^]_0_/mM	[Sn^2+^]_0_/mM	[Pb^2+^]_0_/mM	Ratio
1	10.0	-	-	-	-
2	-	2.0	-	-	-
3	-	-	4.0	-	-
4	-	-	-	0.5	-
5	10.0	2.0	4.0	0.5	20:4:8:1
6	10.0	4.0	4.0	0.5	20:8:8:1
7	20.0	4.0	8.0	1.0	20:4:8:1

## Data Availability

The datasets generated during and/or analyzed during the current study are available from the corresponding authors upon reasonable request.

## Supplementary Materials

The following supporting information can be downloaded at: https://www.mdpi.com/article/10.3390/molecules30061323/s1, Figure S1: The sketch of the experimental setup. Syringes filled with HCl and NaOH stock solutions containing metal salts (1) were placed in programmable syringe pumps (P1, P2). The stock solutions were pumped through Tygon tubes (2) into a quartz cuvette (volume of *V* = 14 mL and optical length of *l* = 2 cm; the constant volume in the cuvette was 8.5 mL) placed in a UV-vis spectrophotometer (3). pH change was measured in real time with a calibrated glass electrode (4) connected to an amplifier (A) and a multimeter (MM). The volume of the reaction mixture was kept constant with a peristaltic pump (P3) and stirred with a magnetic stirrer (5). Pc1–Pc2 were computers for inflow setup and measurements, respectively. Figure S2: Turbidity oscillations in various metal cations mixtures generated in the CSTR by a non-autonomous pH oscillator ([HCl]_0_ = 300.0 mM and [NaOH]_0_ = 300.0 mM): (a) [Zn^2+^]_0_ = 4.0 mM, [Al^3+^]_0_ = 20.0 mM, (b) [Zn^2+^]_0_ = 4.0 mM, [Sn^2+^]_0_ = 4.0 mM, (c) [Zn^2+^]_0_ = 4.0 mM, [Pb^2+^]_0_ = 0.5 mM, (d) [Sn^2+^]_0_ = 2.0 mM, [Pb^2+^]_0_ = 0.5 mM, (e) [Pb^2+^]_0_ = 0.5 mM, [Al^3+^]_0_ = 20.0 mM, (f) [Pb^2+^]_0_ = 0.5 mM, [Al^3+^]_0_ = 10.0 mM; [Sn^2+^]_0_ = 2.0 mM, (g) [Pb^2+^]_0_ = 0.5 mM, (h) [Al^3+^]_0_ = 20.0 mM, [Sn^2+^]_0_ = 2.0 mM. Generated non-autonomous pH oscillations in the CSTR due to the sinusoidally modulated antiphase inflows of acid and base ([HCl]_0_ = 300.0 mM and [NaOH]_0_ = 300.0 mM). Figure S3: Determination of the main characteristic physical quantities in the turbidity peak. Figure S4: Calculated peak width and asymmetry in the turbidity oscillations of aluminum (III), zinc (II), tin (II), and lead (II) metal hydroxides and mixture of aluminum (III) and tin (II) metal hydroxides. Figure S5: Non-autonomous turbidity oscillations in (a) copper (II) hydroxide and (b) zinc (II) hydroxide systems using ammonia solution for the base. (c) Generated non-autonomous pH oscillations in the CSTR due to the sinusoidally modulated antiphase inflows of acid and base ([HCl]_0_ = 300.0 mM and [NH_3_]_0_ = 300.0 mM). Figure S6: Photographs of the patterns in aluminum (III), zinc (II), tin (II), and lead (II) metal hydroxides and their mixtures in agarose gel (1% *w*/*w*). Figure S7: The position of the front in time emerged in a thin tin (II) hydroxide precipitation layer. The front traveled perpendicular to the planar chemical front of the outer electrolyte, moving downward in the gel disk. The solid agarose gel (0.5% *w*/*w*) contained 30.0 mM of tin (II), and a sodium hydroxide solution of 2.500 M was layered on the top of the gel in the Petri dish. Video S1: Propagation of the segmented precipitation zone with a thickness of 70 μm in a confined liquid layer (HS cell) containing 30.0 mM of tin (II), and a sodium hydroxide solution of 2.500 M was used as an outer electrolyte. The width of the frame is 4.2 mm, and the video is 4 times faster than the real process.
