# Supplementary material for: A Non-Autonomous Amphoteric Metal Hydroxide Oscillations and Pattern Formation in Hydrogels
Source: Molecules. 2025 Mar 15;30(6):1323. doi: 10.3390/molecules30061323 (PMC11944573; doi:10.3390/molecules30061323)
Supplement: Supplementary file 1 [file molecules-30-01323-s001.zip › molecules-3479329-supplementary.pdf]

Supplementary Information

A Non-Autonomous Amphoteric Metal Hydroxide Oscillations and Pattern Formation in Hydrogels

Norbert Német 1,2,*, Hugh Shearer Lawson 1, Masaki Itatani 1, Federico Rossi 3, Nobuhiko J. Suematsu 4,5, Hiroyuki Kitahata 6 and István Lagzi 1,7,*

1 Department of Physics, Institute of Physics, Budapest University of Technology and Economics, Műegyetem rkp. 3, H-1111 Budapest, Hungary; lawfia5@gmail.com (H.S.L.); masakiitatani.chem@gmail.com (M.I.)
2 Department of Organic Chemistry and Technology, Budapest University of Technology and Economics, Műegyetem rkp. 3, H-1111 Budapest, Hungary
3 Department of Physical Sciences, Earth and Environment, University of Siena, Piazzetta Enzo Tiezzi 1, 53100 Siena, Italy; federico.rossi2@unisi.it
4 Meiji Institute of Advanced Study of Mathematical Sciences (MIMS), Meiji University, 4-21-1 Nakano, Tokyo 164-8525, Japan; suematsu@meiji.ac.jp
5 Graduate School of Advanced Mathematical Sciences, Meiji University, 4-21-1 Nakano, Tokyo 164-8525, Japan
6 Graduate School of Science, Chiba University, Yayoi-cho 1-33, Inage-ku, Chiba 263-8522, Japan; kitahata@chiba-u.jp
7 HUN-REN–BME Condensed Matter Physics Research Group, Budapest University of Technology and Economics, H-1111 Budapest, Hungary
* Correspondence: nemet.norbert@ttk.bme.hu (N.N.); lagzi.istvan.laszlo@ttk.bme.hu (I.L.)

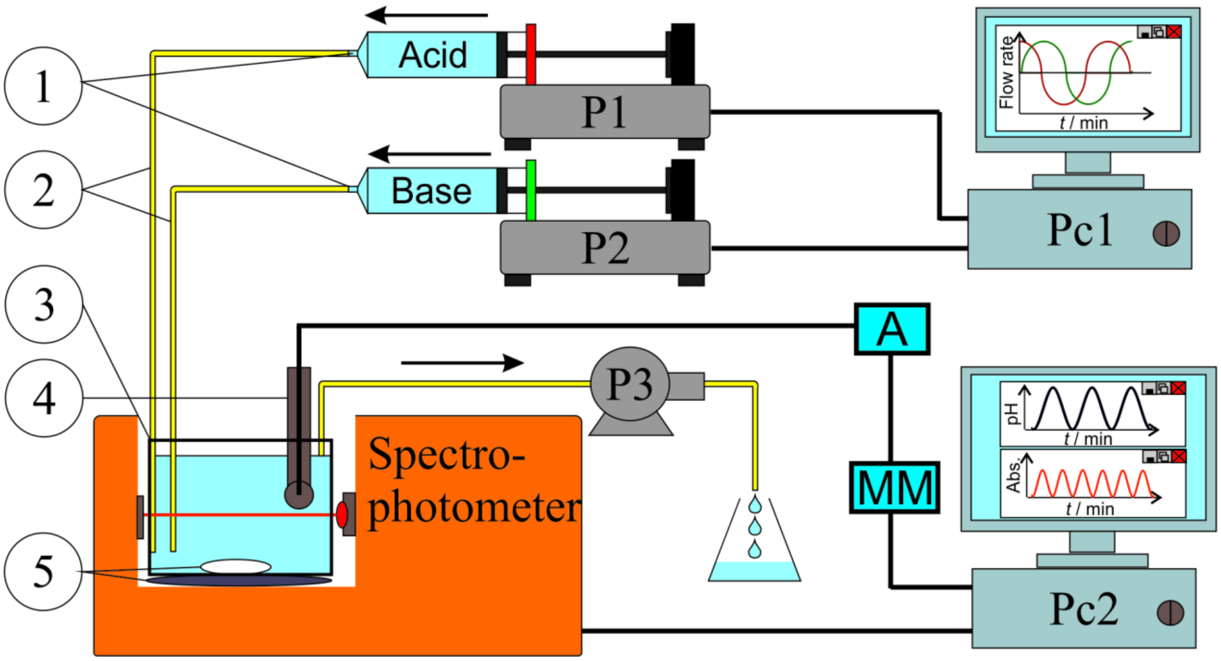

Figure S1: The sketch of the experimental setup. Syringes filled with HCl and NaOH stock solutions containing metal salts (1) were placed in programmable syringe pumps (P1, P2). The stock solutions were pumped through Tygon tubes (2) into a quartz cuvette (volume of $V = 14$ mL and optical length of $l = 2$ cm; the constant volume in the cuvette was 8.5 mL) placed in a UV-vis spectrophotometer (3). pH change was measured in real time with a calibrated glass electrode (4) connected to an amplifier (A) and a multimeter (MM). The volume of the reaction mixture was kept constant with a peristaltic pump (P3) and stirred with a magnetic stirrer (5). Pc1-Pc2 were computers for inflow setup and measurements, respectively.

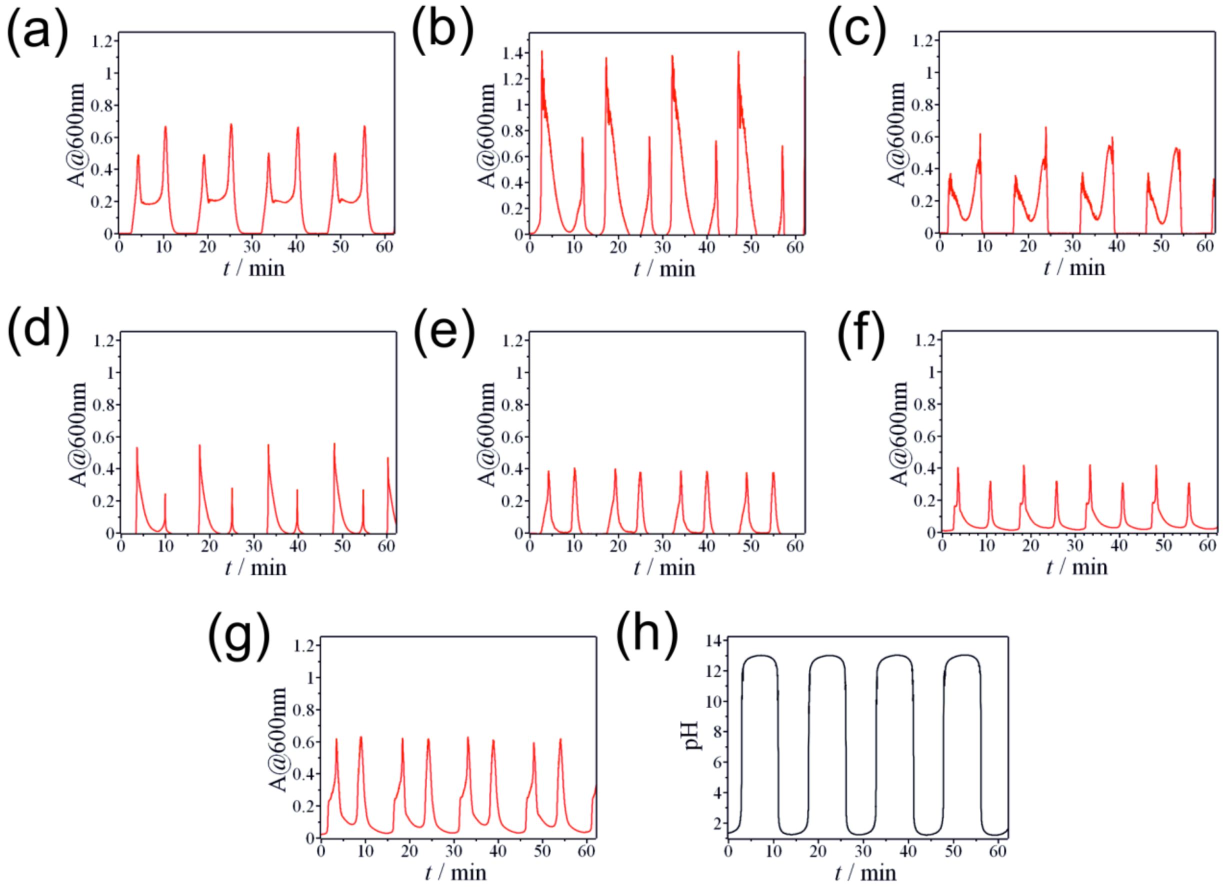

Figure S2: Turbidity oscillations in various metal cations mixtures generated in the CSTR by a non-autonomous pH oscillator ($[HCl]_0$ = 300.0 mM and $[NaOH]_0$ = 300.0 mM): (a) $[Zn^{2+}]_0$ = 4.0 mM, $[Al^{3+}]_0$ = 20.0 mM, (b) $[Zn^{2+}]_0$ = 4.0 mM, $[Sn^{2+}]_0$ = 4.0 mM, (c) $[Zn^{2+}]_0$ = 4.0 mM, [Pb2+]0 = 0.5 mM, (d) $[Sn^{2+}]_0$ = 2.0 mM, $[Pb^{2+}]_0$ = 0.5 mM, (e) $[Pb^{2+}]_0$ = 0.5 mM, $[Al^{3+}]_0$ = 20.0 mM, (f) $[Pb^{2+}]_0$ = 0.5 mM, $[Al^{3+}]_0$ = 10.0 mM; $[Sn^{2+}]_0$ = 2.0 mM, (g) $[Pb^{2+}]_0$ = 0.5 mM, $[Al^{3+}]_0$ = 20.0 mM, $[Sn^{2+}]_0$ = 2.0 mM. (h) Generated non-autonomous pH oscillations in the CSTR due to the sinusoidally modulated antiphase inflows of acid and base ($[HCl]_0$ = 300.0 mM and $[NaOH]_0$ = 300.0 mM).

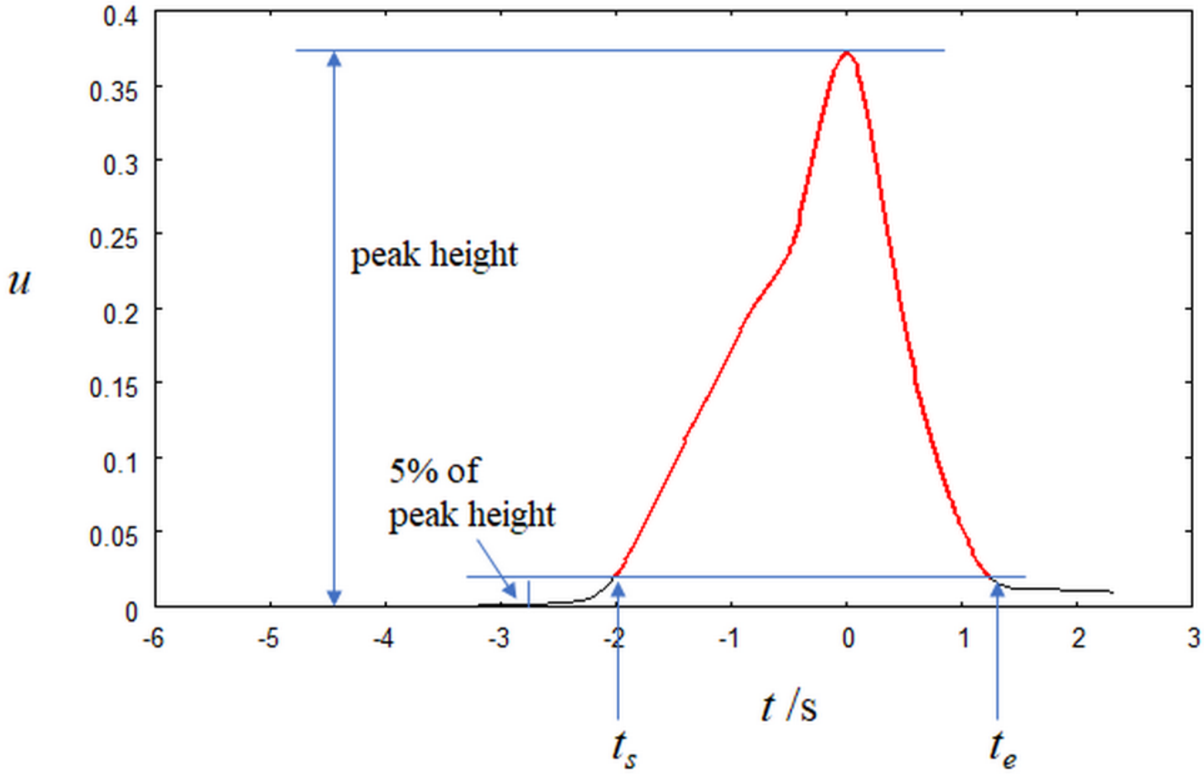

Figure S3: Determination of the main characteristic physical quantities in the turbidity peak.

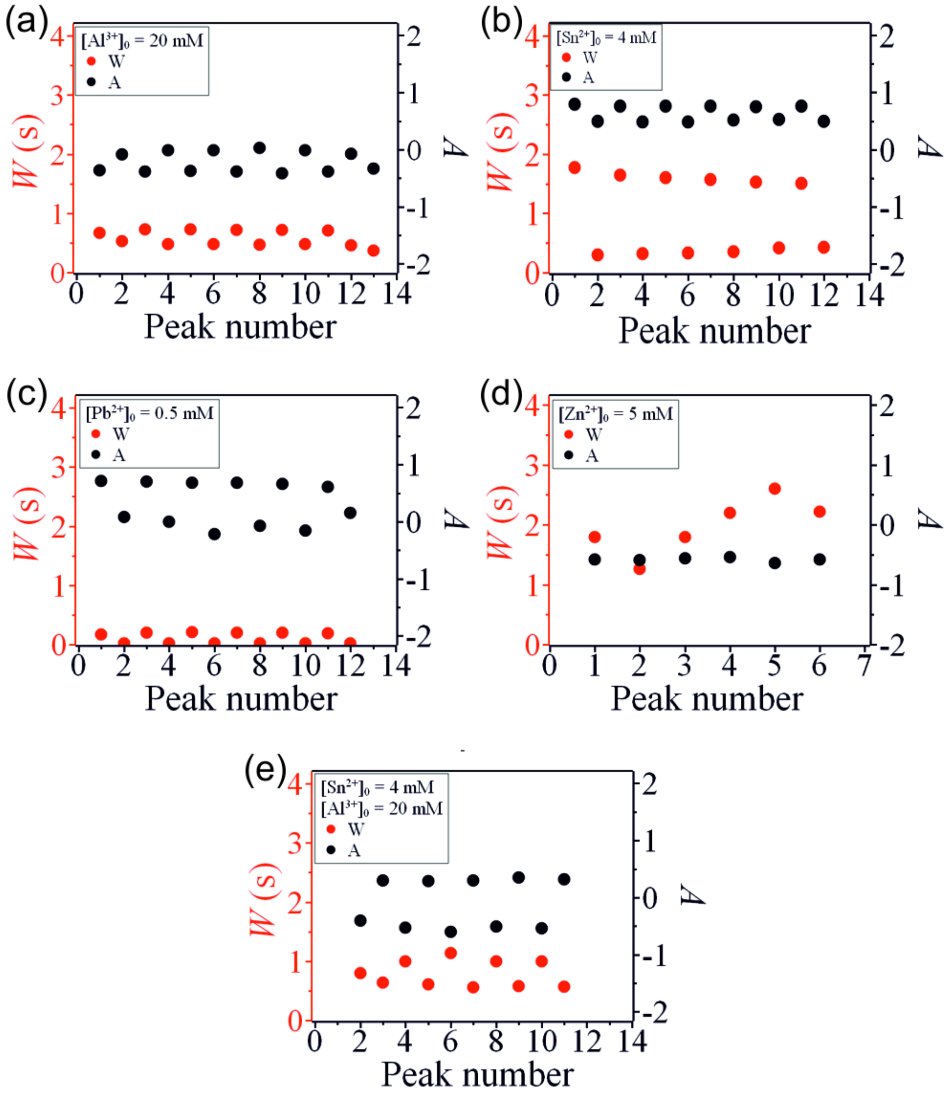

Figure S4: Calculated peak width and asymmetry in the turbidity oscillations of aluminum(III), zinc(II), tin(II), and lead(II) metal hydroxides and mixture of aluminum(III) and tin(II) metal hydroxides.

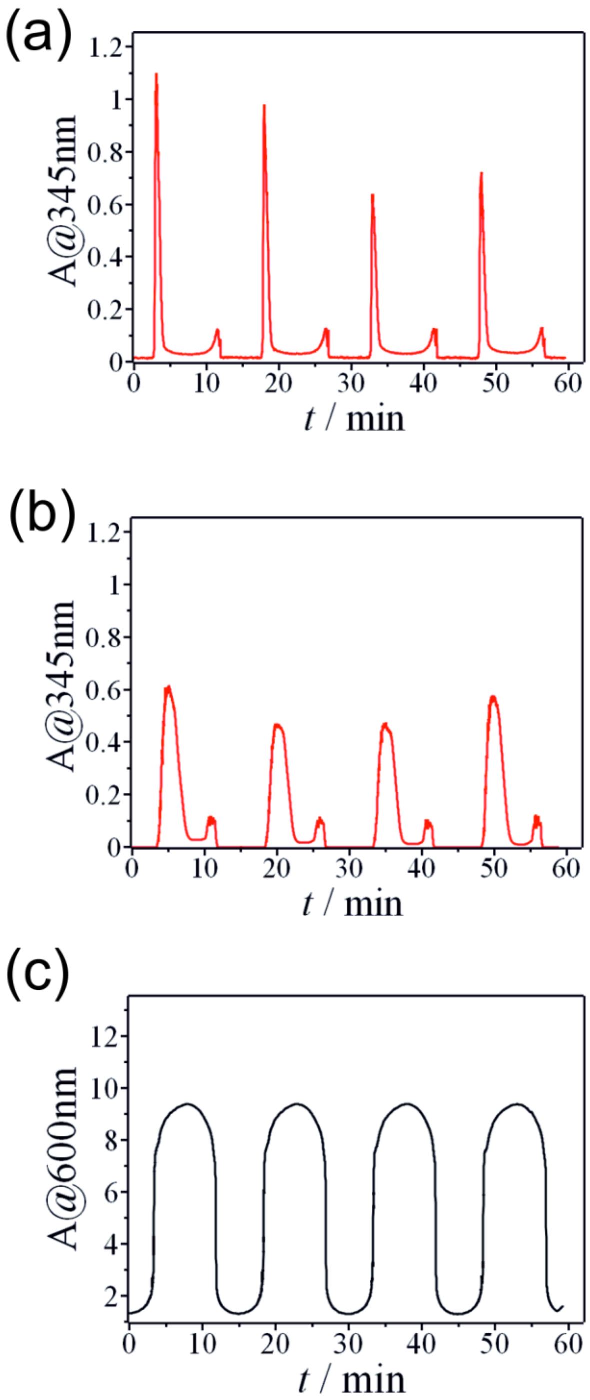

Figure S5: Non-autonomous turbidity oscillations in (a) copper(II) hydroxide and (b) zinc(II) hydroxide systems using ammonia solution for the base. (c) Generated non-autonomous pH oscillations in the CSTR due to the sinusoidally modulated antiphase inflows of acid and base ($[HCl]_0$ = 300.0 mM and $[NH_3]_0$ = 300.0 mM).

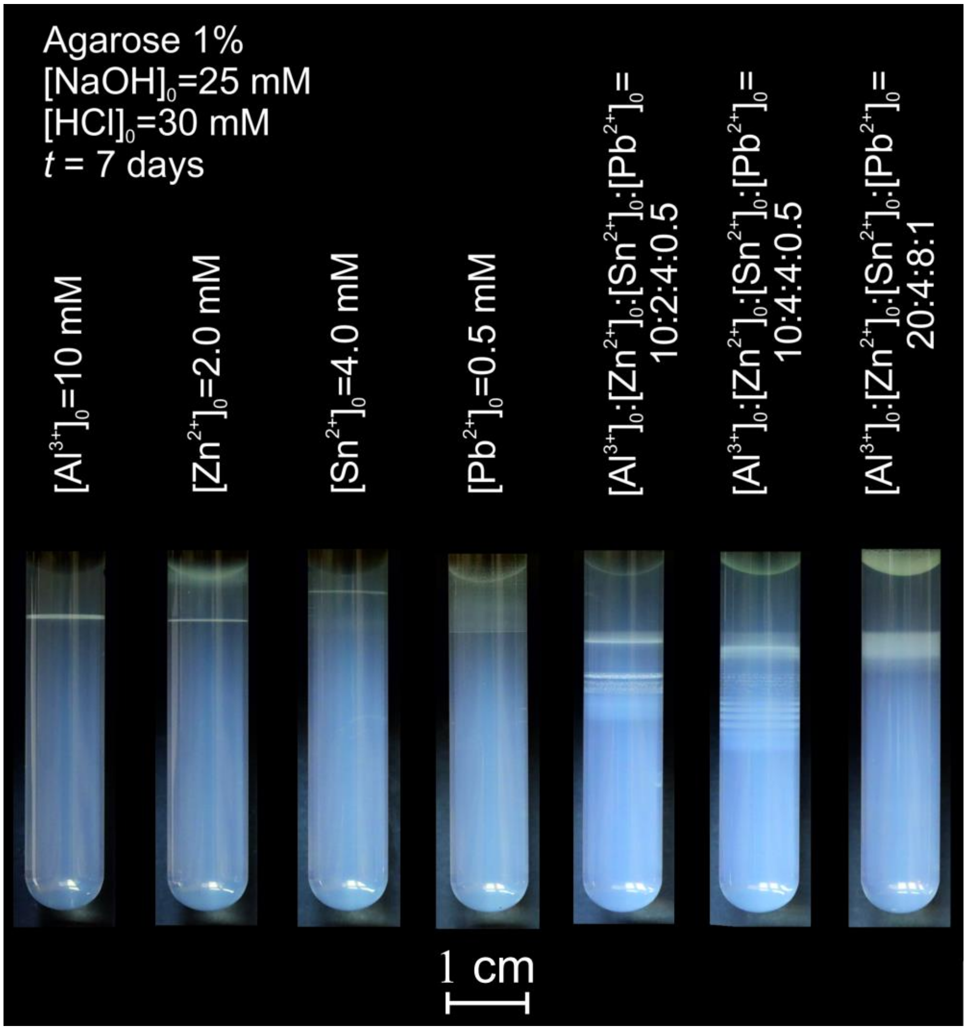

Figure S6: Photographs of the patterns in aluminum(III), zinc(II), tin(II), and lead(II) metal hydroxides and their mixtures in agarose gel (1% w/w).

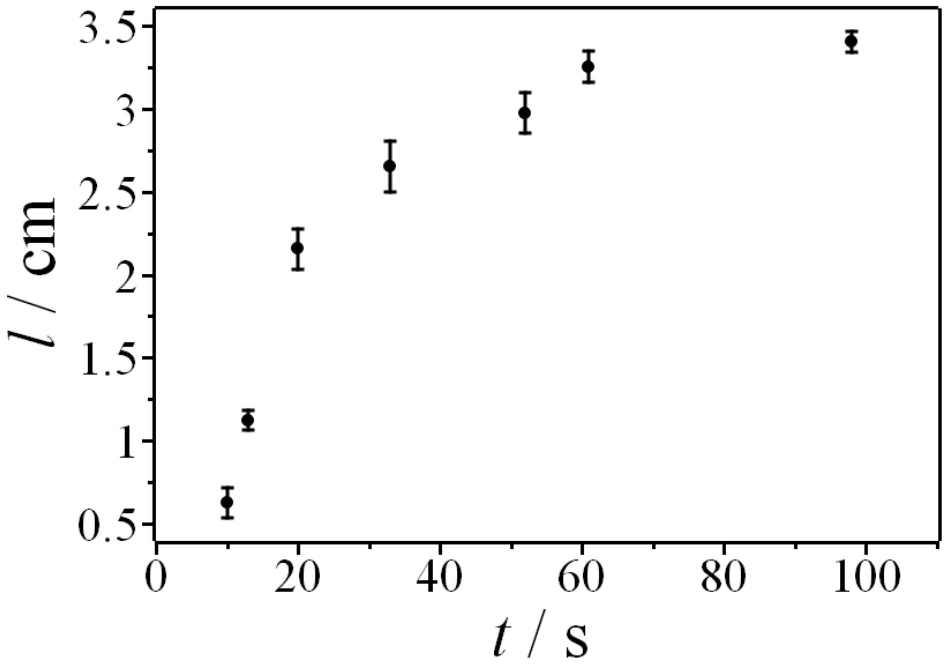

Figure S7: The position of the front in time emerged in a thin tin(II) hydroxide precipitation layer. The front traveled perpendicular to the planar chemical front of the outer electrolyte, moving downward in the gel disk. The solid agarose gel (0.5% w/w) contained 30.0 mM of tin(II), and a sodium hydroxide solution of 2.500 M was layered on the top of the gel in the Petri dish.
